# Oneida County Medical Society

**Published:** 1873-03

**Authors:** 


Oneida County Medical Society.—We present to our readers in this number of the Journal a letter from a member of the Oneida County Medical Society, which, we think, will clearly explain the position of the Society and its opinion in regard to consulting with "all legally qualified physicians." The circular sent us had all the appearance of an official document, and on hasty reading we formed the opinion that the resolutions had the sanction of the Society. We are glad, however, to have the error corrected, and to have the action of the Society fully explained.

